# Dr. Thomas W. Evans

**Published:** 1871-12

**Authors:** 


*Dr. Thomas W. Evans.*—Among the promotions in the Legion of Honor included in the degree published in the *Journal officiel* on Tuesday last, is that of our countryman, Dr. Thomas W. Evans to the rank of commander. "The doctor has been long a member of the order, having been named Chevalier in July, 1853, and Officer in January, 1866. If we do not mistake he is the first foreigner who has obtained the rank of Commander. By the terms of the degree he received his promotion for services rendered as "Director of the American Ambulance," bnt it migth also have been added as its founder. We are glad for two reasons that the Republican Government of France has conferred this distinction on Dr. Evans, first because it has been merited by hard service and large pecuniary sacrifices, and secondly because it is a complete answer to the calumny that he is an Imperialist in his political tendeucies. The Doctor has shown his gratitude to the country which has given him a hospitable reception, by abstaining from all interference in its political affairs. Besides his profession places him in a position so delicate and difficult that it would be impossible for him to gratify such inclinations if he had them.

His course in regard to the Empress, when in her misfortunes she threw herself on his protection, was that of a gallant gentleman and nothing more, and the Government of M. Thiers fully recognizes the fact by conferring upon him a distinction, which, though unsought for, has been earned by zealous and substantial service to France."

